# Work Smarter Not Harder: Mapping Interprofessional Education Collaboration Core Competencies Across Curricula

**DOI:** 10.1007/s40670-025-02337-4

**Published:** 2025-03-04

**Authors:** Hanlie Pitout, Paula Barnard-Ashton, Fasloen Adams, Sanetta H. J. du Toit

**Affiliations:** 1 Department of Occupational Therapy, Sefako Makgatho Health Sciences University, Pretoria, South Africa; 2 Teaching and Learning Office, Faculty of Health Sciences, Department of Occupational Therapy, University of Witwatersrand, Johannesburg, South Africa; 3 Division of Occupational Therapy, Department of Health and Rehabilitation Sciences, Stellenbosch University, Stellenbosch, South Africa; 4 Occupational Therapy, School of Medical and Health Sciences, Edith Cowan University, 270 Joondalup, Joondalup, 6027 Australia

**Keywords:** Curriculum mapping, IPEC core competencies, Undergraduate curriculum, Curriculum analysis, Health professions education

## Abstract

Integrating Interprofessional Education and Collaborative Practice (IPECP) into health professions curricula is recognized as a challenging task due to factors such as timing, curriculum overload, and resource availability. This study aimed to identify opportunities within courses across all health professions that would help students achieve the Interprofessional Education Collaborative (IPEC) Core Competencies. These competencies include (1) Values and Ethics, (2) Roles and Responsibilities, (3) Interprofessional Communication, and (4) Teams and Teamwork. A quantitative, cross-sectional document review was conducted, analyzing curriculum maps and documents from 11 healthcare professions at a South African university. This process involved 22 participants, comprising two representatives from each profession who possessed expertise in the content and teaching methods relevant to their field. They completed a structured survey using the REDCap platform, with guidance from the first author, who was knowledgeable about the IPEC competencies. The results indicated that the curricula of all professions incorporated the IPEC Core Competencies. Notably, the Values and Ethics competencies had the highest representation (mean = 101.2), while Teams and Teamwork were the least represented (mean = 64.6). Thus, the analysis of the curriculum maps provided valuable insights for curriculum planners, allowing them to identify gaps and overlaps. This information serves as a foundation for developing an integrated, longitudinal, evidence-based IPECP curriculum.

## Introduction

Interprofessional Education and Collaborative Practice (IPECP) should be prioritized, but it is often viewed as an “add-on” to overcrowded profession-specific curricula. Students and lecturers tend to value profession-specific content over IPECP opportunities [1, 2]. Implementing IPECP involves learning with, from, and about peers in educational settings and applying this knowledge during work-integrated experiences with various health professionals [3].

There is an ongoing debate about the best delivery system for IPECP curricula, with no one-size-fits-all solution [4–6]. Integrating IPECP effectively within a competency-based educational approach remains challenging for university management, lecturers, and students [4–6].

International frameworks such as the Interprofessional Capability Framework (UK), the National Interprofessional Competency Framework (Canada), and the Core Competencies for Interprofessional Collaborative Practice (US) guide interprofessional education [7]. This study uses the Interprofessional Education Collaborative’s Core Competencies for Interprofessional Collaborative Practice (IPEC) [8]. The four main IPEC Core Competencies are Values and Ethics (VE), Roles and Responsibilities (RR), Interprofessional Communication (IC), and Teams and Teamwork (TT). An updated 2023 IPEC version became available after the data collection was done [9]. The main difference between the 2016 and 2023 versions is the formulation of the Sub-competencies, while the four Core Competencies stay the same. Therefore, the results refer to the 2016 formulation of Sub-competencies (see the Appendix Table 3).

Using IPEC Core Competencies in curriculum planning promotes a common language and clarity, encourages collaboration among educators, and ensures relevance to practice [6]. Mapping these competencies to learning objectives helps shift the focus from knowledge transfer to skill development, allowing for the identification of common competencies across professions [4, 10, 11].

Curriculum mapping, originally introduced by English [12], and applied to interprofessional education and collaborative practice (IPECP) in various studies [13–15], can significantly aid in identifying, developing, reviewing, improving, and refining IPECP curricula [16, 17]. It serves as a valuable tool for detecting gaps and potential overlaps within these curricula [18, 19].

The advantage of a curriculum map lies in its presentation of curriculum information from multiple perspectives, highlighting the relationships between key elements such as courses, units, learning outcomes, and assessments [20]. When curriculum planners analyze this information across different professions, they can better visualize the overlaps among them, facilitating the effective integration of IPECP into a university’s professional programs).

When engaging in curriculum mapping, it is essential to ensure that the IPECP experience promotes equality and mutual respect for knowledge among the various professions involved [17]. Additionally, the IPECP curriculum should be integrated throughout degree programs rather than treated as a standalone or compulsory addition in a specific year of study [21]. Integrating an IPECP curriculum with existing professional curricula could be a valuable solution for universities that currently lack an IPECP component or wish to establish comprehensive IPECP engagement across all years of undergraduate study.

The larger study, published elsewhere, includes a phase focused on curriculum mapping. The overall aim was to develop an integrated and comprehensive IPECP curriculum for the university. This curriculum mapping phase specifically aims to identify optimal opportunities for developing IPEC Core Competencies that can be integrated across 11 existing undergraduate programs. These identified opportunities should facilitate collaborative and longitudinal learning.

The objectives of this phase include analyzing the curriculum map to identify:

1. The specific year in which IPEC Sub-competencies are addressed within each profession’s curricula.
2. The gaps in IPEC Core Competencies within the profession’s curricula.
3. The priority IPEC Sub-competencies for undergraduate students.
4. The existing IPEC Sub-competencies already present in the profession’s degree programs, to serve as the foundation for the IPECP curriculum.

## Method

The overarching research question was.

What would support an evidence-based foundation for the development of an integrated IPECP curriculum that could use resources more effectively to prepare practice-ready graduates of the 11 involved professions?

### Study Context

The study was conducted at a public university in South Africa, involving four health science schools in Interprofessional Education and Collaborative Practice (IPECP). The participating undergraduate programs included Occupational Therapy, Physiotherapy, Human Nutrition and Dietetics, Speech Language Pathology and Audiology, Nursing, Medicine, Radiography, Pharmacy, Dentistry, Oral Hygiene, and Dental Therapy. Since 2019, their curricula have been mapped on the Learning Opportunities, Objectives and Outcomes Platform (LOOOP) [22]. LOOOP is a web-based platform that identifies overlaps and gaps in program content [23–25]. LOOOP maps to different competency frameworks including CanMEDS Core Competencies (this is a framework to indicate the abilities required to effectively meet the health care needs), Medical Subject Headings [26], National Qualifications Framework (NQF) of South Africa (NQF is a system that sets requirements and guidelines for specific levels of qualification), and Critical Crossfield Outcomes and profession-specific exit level outcomes (that is skills or competencies that are considered essential for success across different fields). LOOOP allows for instant mapping across all professions and courses. However, at the time of data collection, LOOOP did not include an IPEC competency framework so data on Core Competencies and Sub-competencies could not readily be extracted.

In 2019, the university launched a 1-week IPECP pilot event for final-year students from six professions, which expanded to 11 professions by 2023, with support from faculty and administration. However, disparities in event duration and student enrolment complicated the pilot. For instance, in 2022, a group of 27 students included 11 from medicine, primarily sixth-year students, while most others were in their fourth year.

These differences necessitated a re-evaluation of the IPECP program’s implementation to avoid rivalry and ensure learning effectiveness. It is crucial to match IPECP learning opportunities to students’ levels of knowledge and skills in future curriculum updates [27, 28].

### Research Design

This study utilized a quantitative, cross-sectional document review [29] to analyze the curriculum maps of 11 undergraduate health professions. The aim was to identify the Core Competencies and Sub-competencies that were addressed in each profession and each year as well as common topics across existing curricula as a foundation for developing an integrated Interprofessional Education and Collaborative Practice (IPECP) curriculum. The analysis was guided by the 2016 IPEC document, although the updated 2023 version refines the Sub-Competencies. Despite these updates, the four main categories remain consistent between the 2016 and 2023 versions.

### Data Collection Instrument

An electronic data collection instrument was developed using Research Electronic Data Capture software (REDCap) [30]. REDCap is a secure online platform specifically designed for individualized instrument design, data collection, and data management. This instrument was employed to gather data from each of the 11 professions.

The instrument consisted of two digital forms: a general curriculum information form and linked course-specific forms for each course in the profession. The general curriculum information form collected data on the duration of study in terms of years, and the allocated credits based on notional hours of study for the professional undergraduate degree. The course-specific forms gathered information on several aspects, including the number of credits, year level, whether the course was profession-specific, and the details related to the IPEC Core Competencies and Sub-competencies. Courses that incorporate core competencies are referred to as “core competency courses,” which represent a subset of courses in each profession that develop foundational knowledge of the IPEC core competencies.

Below is a screenshot from a section of the data collection instrument, specifically illustrating the type of data collected from the course-specific form.
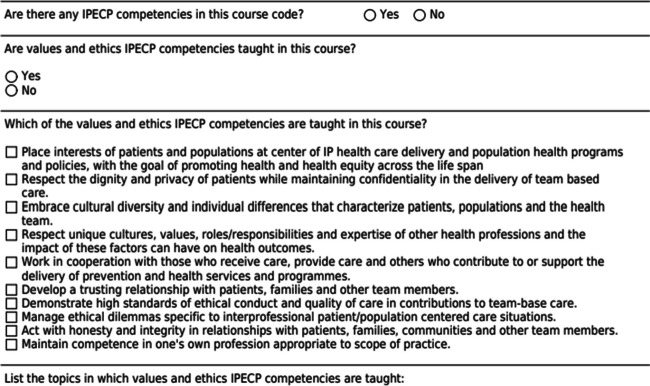


### Data Collection Process

Two faculty representatives from each profession participated in a quantitative, cross-sectional document review alongside the principal investigator (first author). They used a data collection instrument on REDCap to complete the review. The documents reviewed included LOOOP information, learner guides, and course data found in official institutional documentation.

Both faculty representatives were familiar with the teaching methods, course content, specific courses, and objectives, which enabled them to identify the IPEC Core Competencies addressed in their respective courses. One representative was a profession-appointed member of the university’s IPECP committee, responsible for implementing existing IPECP activities. The second representative was part of the school curriculum committee, which oversees curriculum matters for their profession and was involved in the LOOOP curriculum mapping process. The principal investigator guided the representatives in understanding the IPEC competencies and specific sub-competencies. A standardized data collection procedure was followed: representatives were briefed on the purpose of the document analysis, the Core Competencies and Sub-competencies were explained, and the Core Competency document was used as a reference throughout the review process. Documents were selected based on the criterion that they provided the most recent information about the courses. A pilot review of the documents from one profession was conducted to identify any potential flaws before the main analysis. For data validation, understanding among all three participants was verified before capturing the data on the REDCap form. Data from the 11 involved professions were verified by comparing information provided by representatives, who consulted with course experts to confirm Sub-competencies, reviewing information available on LOOOP, and checking details in school calendars. Course objectives and teaching methods were main source of information to identify the specific Sub-competencies that were addressed.

### Data Analysis

Descriptive statistics were utilized to analyze data collected through the REDCap data collection instrument. The data were exported from REDCap to Microsoft Excel for further analysis. The general curriculum information for each profession was compared, and the course-specific data were examined using Excel to summarize a large volume of information. This analysis focused on extracting variables such as profession, year of study, and specific sub-competencies.

Additionally, IPEC Core Competencies were analyzed based on the percentage of sub-competencies present within each core competency. This analysis sought to identify which IPEC Core Competencies and associated sub-competencies were most or least incorporated across different years and professions in the professional curricula.

## Results

The most significant finding is that the curricula for all professions incorporated all four IPEC Core Competencies, with the Values and Ethics competency being the most prominently represented. In contrast, Teams and Teamwork were the least integrated competencies.

### Professions and Core Competencies Incorporated in Curricula

Table 1 summarizes the various professions from the general curriculum form, including the number of courses, credits, and years of study. A total of 356 courses were offered across 11 professions, of which 195 (54.8%) were identified as core competency courses. Although these courses were not taught in an interprofessional manner, it was noted that they addressed the IPECP Core Competencies. In six of the 11 professions, there are less than 50% core competency courses. Seven of the professions have a 4-year course for which only 480 credits (120 per year) are subsidized, indicating that the majority of professions are exceeding the number of subsidized credits.

**Table 1:** Overview of the professions and core competency courses

School	Profession	Overall credits	Number of years of study	Number of courses in the profession	Number of core competency courses (%)
Health Care Sciences	Occupational Therapy	480	4	28	22 (78.6)
	Nursing	512	4	37	22 (59.5)
	Human Nutrition and Dietetics	512	4	28	20 (71)
	Physiotherapy	512	4	20	12 (60)
	Speech Language Pathology and Audiology	512	4	55	21 (38)
Medicine	Radiography	540	4	28	13 (46)
	Medicine	920	6	41	23 (49)
Oral Health Sciences	Dentistry	804	5	41	19 (46)
	Dental Therapy	356	3	26	12 (46)
	Oral Hygiene	364	3	27	11 (41)
Pharmacy	Pharmacy	502	4	25	20 (80)

Three core competency courses were common to all professions: English for Health Professionals, Psychology, and Research Methodology. Although there were several other common courses, such as Anatomy, Basic Sciences, and Pharmacology, representatives agreed that these courses did not incorporate IPEC Core Competencies and therefore were not classified as core competency courses.

Pharmacy and Occupational Therapy reported that the majority of their courses were core competency courses, with 20 (80%) and 22 (78.6%) of their courses falling into this category, respectively. In contrast, Speech Language Pathology and Audiology reported that only 21 out of 55 courses (38%) were classified as core competency courses.

### IPEC Core Competency Representation

**Fig. 1:**
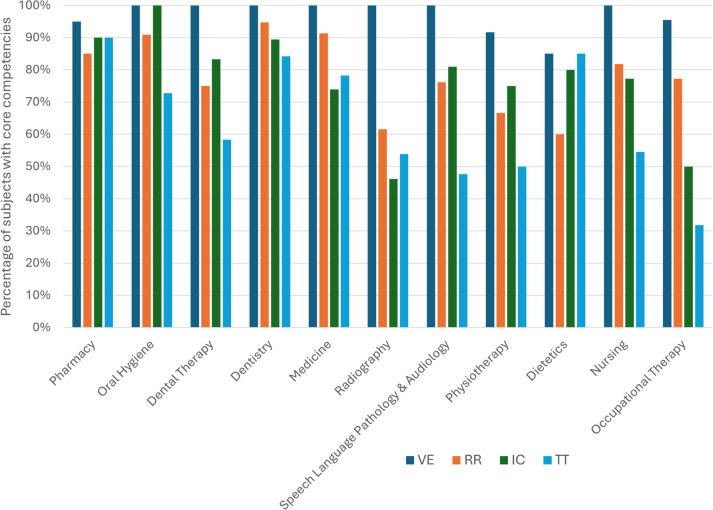
Core competency courses per profession

Figure 1 shows that the Core Competency most commonly included across the 11 professions is Values and Ethics (VE), which appears in the highest percentage of core competency courses. In contrast, the Teams and Teamwork (TT) Core Competency is the least represented among the four Core Competencies in seven of the professions. The information in Fig. 1 illustrates how well the students in each profession are prepared regarding IPEC competencies.

### IPEC Sub-Competency Representation

Table 2 summarizes the distribution of IPEC core competencies across each year. The mean and range for each IPEC Core Competency are detailed in the Appendix, with a more comprehensive analysis available upon request. The inclusion of both the range and the percentage allows for a meaningful comparison among the Core Competencies, as they do not all consist of the same number of sub-competencies.

**Table 2:** The representation of core competency courses per IPEC Core Competency and number of professions per year

Number of professions per year	Values and Ethics		Roles and responsibilities		Interprofessional communication		Teams and teamwork	
	Mean	Range (%)	Mean	Range (%)	Mean	Range (%)	Mean	Range (%)
Year 1 (*n* = 11)	6.5	1–11 (9–100)	3.5	0–10 (0–91)	7.5	1–11 (9–100)	1.5	0–3 (0–27)
Year 2 (*n* = 11)	8.3	1–11 (9–100)	5.7	1–1 (9–100)	6.6	4–10 (36–91)	4.7	1–8 (9–73)
Year 3 (*n* = 11)	9.4	2–11 (18–100)	8.1	2–11 (18–100)	8.4	6–11 (55–100)	7.4	3–10 (27–91)
Year 4 (*n* = 9)	7.9	3–9 (33–100)	7.2	3–9 (33–100)	7.3	6–8 (67–89)	6.5	2–9 (22–100)
Year 5 (*n* = 2)	2.0	2–2 (100–100)	1.3	0–2 (0–100)	2.0	2–2 (100–100)	1.5	0–2 (0–100)
Year 6 (*n* = 1)	1.0	1–1 (100–100)	1.0	1–1 (100–100)	1.0	1–1 (100–100)	0.7	0–1 (0–100)
Overall	101.2	21–128 (11–66)	70.5	(11–144 (6–74)	92.9	62–130 (32–67)	64.6	15–90 (8–46)

According to Table 2, the Values and Ethics competency was the most represented among the four Core Competencies, with a high overall number of courses incorporating its sub-competencies (mean = 101.2). In contrast, the Teams and Teamwork competency had the lowest representation, with a mean of 64.6.

### IPEC Core Competency Development Across Years of Study

**Fig. 2:**
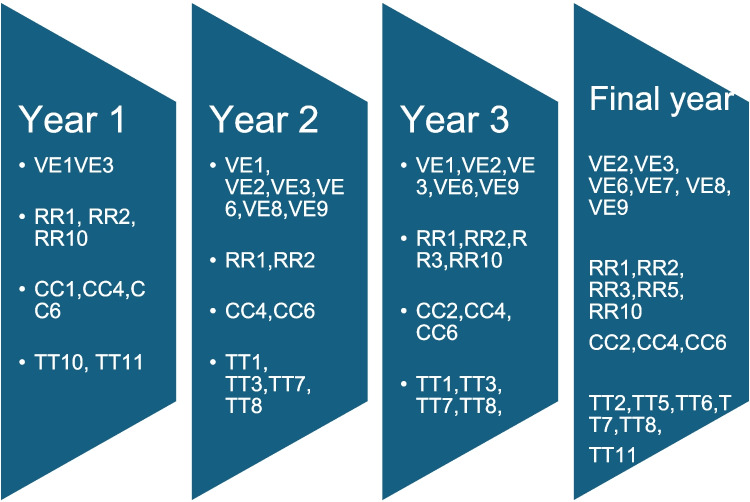
IPEC Core Competency development across years of study

Figure 2 illustrates the development of IPEC Sub-competencies over the years. Each year has introduced new Sub-competencies, while also maintaining those from previous years, although the earlier Sub-competencies have become more refined. Table 2 confirms that the Core Competencies addressed increased from the first to the fourth year, with only limited additions in the fifth and sixth years. Since most professions require a 4-year undergraduate degree, four levels were identified, with the fourth level representing the final year for all professions. For professions with 3-, 5-, or 6-year degrees, they determine how their students integrate into the curriculum; for instance, students in medicine and dentistry attend classes with peers from other professions during their first three years, and in their final years, they join the fourth-year student cohort.

## Discussion

The curriculum analysis revealed opportunities for Interprofessional Education and Collaborative Practice (IPECP) by identifying both overlaps and gaps in the curricula of various professions concerning the IPEC Core Competencies. Recognizing these overlaps and gaps is a significant advantage of curriculum mapping [18, 31].

A comprehensive analysis of the inclusion of specific Sub-competencies based on the 2016 IPEC Core Competencies is provided. The foundation of the IPEC competencies, established by mapping these competencies to the courses within the current professions’ curricula, equips students with the essential skills needed to work effectively as part of a team.

Currently, in the curricula of these professions, students lack the opportunity to practice working alongside a variety of professionals, each bringing unique contributions to patient care. By embedding an interprofessional curriculum within the existing professions’ curricula, we can utilize this integration to help students apply the knowledge they have gained in their specific professions within an interprofessional context. This can occur both during interprofessional education sessions and in clinical settings, where students can collaborate to provide team-based care.

Specific details relating to the IPEC Core Competencies were identified.

### Values and Ethics

Values and Ethics emerged as the predominant Core Competency across all health professions, incorporating these elements in over 80% of core competency courses for each profession. This is an expected outcome, as a key aspect of being a health professional is understanding one’s values and those of patients, while also respecting diversity and promoting health equity [8]. Values and Ethics encompass professionalism and enhance patient and community-centered care, which is vital for delivering quality health services [32]. Ethical practice is essential and recognized as a crucial component of continuous professional development, as unethical behavior can lead to significant legal consequences [33]. In designing an Interprofessional Education and Collaborative Practice (IPECP) curriculum, the widespread recognition of Values and Ethics across professions provides a common foundation for all students to build upon.

There are ten sub-competencies related to Values and Ethics, which are included in a mean of 101.2 core competency courses. Across various years of study, the sub-competencies of valuing diversity [VE]), building trusting relationships [VE6], and exhibiting honesty and integrity in relationships [VE9] were particularly prominent. In an IPECP curriculum, Values and Ethics are greatly influenced by teamwork. Acceptance of individual differences and the establishment of relationships based on trust, honesty, and integrity contribute to developing these relationships from a values-based perspective. This foundation can be taught and practiced within individual professions before applying these skills interprofessionally.

The refinement of most Values and Ethics sub-competencies relies on students’ clinical experiences with patient care, which typically occurs during their later years of study. This is particularly true for competencies related to respecting dignity and privacy (VE2), ethical conduct, and quality care (VE7 and VE8). For instance, the importance of promoting values and interests (VE1) and respecting dignity and privacy (VE2) is incorporated throughout all years and serves as a basis for future learning. Koehn and Charles [34] [42] also noted that participants recognized these competencies as fundamental core values.

The development of a multi-year IPECP curriculum depends on providing sufficient exposure to interprofessional team-based care [32]. Emphasis on recognizing uniqueness and diversity (VE4), as well as collaborating with other team members and recipients of care (VE5), prepares students for effective teamwork. The low representation of maintaining competence (VE10) is expected, as students are still in the process of developing their skills.

### Roles and Responsibilities

The Roles and Responsibilities Core Competency is generally well integrated across various professions; however, it is the least evident in Human Nutrition and Dietetics (present in 60% of 20 core competency courses) and Pharmacy (present in 85% of 20 core competency courses). On average, the ten Roles and Responsibilities Sub-competencies are evident in about 70.5% of the core competency courses.

The Sub-competencies related to communication about roles (RR1), recognition of one’s own limitations (RR2), and collaboration (RR10) are demonstrated across most professions and throughout all years of study. These Sub-competencies can be introduced theoretically during the first year of study and expanded upon in later years. On the other hand, Sub-competency RR3, which addresses complementary expertise through engaging with various professions, as well as role clarification (RR4), understanding the full scope of each profession (RR5), and recognizing complementary expertise (RR9), is typically developed in the more senior years. These elements rely heavily on opportunities for interprofessional clinical involvement.

Expectations for competence, such as forging independent relationships (RR7) and committing to continuous professional and interprofessional development (RR8), are not realistic at the undergraduate level.

The Roles and Responsibilities Core Competency begins to take shape in the early years of study, and it is crucial to cultivate both the formation of a student’s professional identity as a member of a unique profession and as part of an interprofessional team through the Interprofessional Education and Collaborative Practice (IPECP) curriculum [35]. O’Keefe et al. [6] emphasize that explaining interprofessional practice to patients, clients, families, and other professionals, akin to RR1, and describing the areas of practice for other health professions, similar to RR4, are among the eight most important competencies to be achieved in interprofessional learning.

### Interprofessional Communication

The Interprofessional Communication Core Competency, which includes eight Sub-competencies, was generally well integrated across various professions. Although it is more prevalent in the first year of study, there is potential for its application in subsequent years. All professions incorporate the courses “English for Health Professionals” and “Psychology” during the first year, which likely ensured that the core competencies of interprofessional communication—such as the use of communication tools, techniques, and technologies (CC1); clear communication (CC2); promoting common understanding and active listening (CC4); and respectful communication (CC6)—were evident across all professions.

Busenhart [36] also found these sub-competencies to be appropriate for novice students. Among the eight competencies, clear communication (CC2) and common understanding (CC3) are essential. O’Keefe et al. [6] emphasized the importance of these competencies for expressing professional opinions competently, confidently, and respectfully, while avoiding profession-specific language.

Core Competencies do not exist in isolation. The development of the Interprofessional Education and Collaboration Practice (IPECP) curriculum should integrate Interprofessional Communication with the learning of Roles and Responsibilities and Teams and Teamwork. For instance, common understanding (CC3) and recognition of one’s uniqueness (CC7) depend on developing the Roles and Responsibilities Core Competencies. Similarly, giving and using feedback (CC5) and communicating the importance of teamwork (CC8) require competencies associated with Teams and Teamwork.

Interprofessional Communication is a competency that necessitates continued learning and reinforcement throughout all years of study [36]. A pragmatic approach to fostering effective Interprofessional Communication could involve using communication tools, techniques, and technologies (CC1), promoting common understanding and active listening (CC4), providing and receiving feedback (CC5), and encouraging respectful communication (CC6). While these elements could be part of basic science courses, they are not typically taught in a way that addresses these sub-competencies. Consequently, basic science courses, such as Anatomy, were not included as core competency courses.

### Teams and Teamwork

The least incorporated core competency among seven professions was Teams and Teamwork, particularly in Occupational Therapy, where only 32% of the 22 core competency courses addressed it. On average, the 11 Teams and Teamwork sub-competencies were present in 64.6% of these core competency courses. There was minimal evidence of these Sub-competencies in the first year across the professions, likely due to the emphasis on foundational knowledge in basic science and profession-specific courses. In the first year, the topics of team development and effective team practices (TT1) and reflection on individual and team performance (TT8) were included. This aligns with Busenhart’s findings [36], which suggested that these sub-competencies are appropriate for beginners.

Most Teams and Teamwork sub-competencies were highlighted during the senior years. Additionally, the sub-competencies of sharing accountability (TT7) and reflecting on individual and team performance (TT8) were the most frequently represented across professions. Conversely, the sub-competencies related to process improvement and organizational structures (TT9 and TT10) were the least integrated at the undergraduate level. This may be attributed to the fact that undergraduate students are primarily focused on treating individual clients rather than considering larger systems and processes. While systems and processes might be covered theoretically in undergraduate courses about service management, they are typically managed at a higher level, such as by department heads or senior managers, in clinical settings.

The first Teams and Teamwork sub-competency (TT1), “Describe the process of team development and the roles and practices of effective teams,” is rarely evident across the years in the professions, yet it is foundational for interprofessional education [37] competence. Professions should be made aware of the need to integrate the knowledge component into their specific training, and the application can then be developed from the first to the final year of IPE.

### Practical Implications for the Development of an IPECP Curriculum

#### Credit-Bearing Courses or Integrated IPECP Curriculum

One important consideration in planning an Interprofessional Education and Collaborative Practice (IPECP) curriculum is whether it should be offered as a separate credit-bearing course or integrated into existing professional curricula. An analysis of the curricula for various professions (see Table 1) revealed that most professions are already exceeding the maximum allowable credits (480 credits for 4 years) subsidized by the Department of Higher Education at this university. In the South African context, subsidized credits refer to the number of credits that a profession is permitted to teach to receive government funding, which may impact or limit the scope of the teaching content. As a result, many professions are hesitant to add a new credit-bearing course, as this may require them to undergo reaccreditation from their statutory bodies—a process that is often lengthy and complicated.

While the number of courses for each profession varies, all 11 professions include core competency courses that encompass content essential for developing the IPECP Core Competencies, ranging from 38 to 80% of the courses in a profession’s curriculum. These 195 core competency courses present opportunities for interprofessional learning, such as ethics. Consequently, this curriculum mapping and analysis study suggests that a more effective method for developing an IPECP curriculum is to embed IPECP content within the existing professional programs, rather than offering it solely as a separate course, which is not a feasible option given the already full curricula.

Curriculum mapping and analysis could serve as a foundation for a longitudinal curriculum, where the application of IPECP is based on content already included in the professions’ curricula. This approach has the added advantage that IPECP assessments can then be incorporated into the assessments of the professions’ courses linked to the content addressed in the IPECP sessions. By repackaging existing course content to integrate IPECP throughout all years of study, rather than presenting it as a separate course, this strategy offers a pragmatic solution that emphasizes working smarter, not harder.

#### IPECP Curriculum Focus on Expanding and Application of Profession-Specific Knowledge

The IPECP curriculum aims to expand and apply profession-specific knowledge by providing students with opportunities to develop and use the Core Competencies needed for interprofessional collaborative practice [8, 9]. Although this study identified where the IPEC Core Competencies are integrated into health profession curricula, instruction tends to be more theoretical, lacking the practical integration needed for effective interprofessional learning [38].

Effective IPECP curriculum planning should focus on both expanding profession-specific knowledge [6] and demonstrating the value of interprofessional skills and attitudes within each profession’s courses. These competencies cannot be fully developed through traditional profession-specific education, which often emphasizes knowledge over practical interprofessional skills and collaboration [6].

#### Prioritizing IPEC Sub-Competencies by Year

Curriculum mapping and analysis allow the identification of the overlap and gaps among various professions and establish the IPECP content for each year. This process reveals the preparedness of students from different professions in the IPEC Sub-competencies. While all IPEC Core Competencies can be addressed, not all Sub-competencies are suitable for undergraduate study [39].

The value of this analysis lies in its ability to prioritize specific Sub-competencies for each academic year. This aligns with Busenhart’s findings [36], showing which competencies can be realistically achieved during different years. Such identification helps shape learning objectives and educational activities and contributes to scaffolding the curriculum [40].

Effective learning transfer occurs when students can connect new information to prior knowledge, enhancing their competence and application skills in real-world scenarios. Demonstrating how each year builds on the previous one is crucial for developing both attitudes and skills in patient-centered care [10, 41]. The focus on specific Sub-competencies for each year, could improve the planning of the IPECP curriculum and align it with profession-specific education.

#### Developing an Evidence Base for Decision-Making

Mapping the curriculum and analyzing how courses incorporate the IPEC Core Competencies creates a framework for evidence-based decision-making in Interprofessional Education and Collaborative Practice (IPECP). This mapping also supports quality assurance during implementation [19].

The IPECP curriculum must ensure coherence across its elements, particularly in showing gradual competence growth through the achievement of IPEC Core Competencies over the years [42]. While some authors [34–36] discuss the development of these competencies from novice to advanced skills for independent practice, this study focuses on specific yearly achievements.

We identified profession-specific and multiprofessional learning opportunities as foundations for interprofessional learning. Thistlethwaite and Moran [43] suggest that a multiprofessional approach—where students learn in parallel—can sometimes be more efficient and resource-effective than interprofessional methods. Courses like Anatomy and Physiology may significantly foster IPEC Core Competencies, as they include students from various professions and can encourage learning with, from, and about each other, however, it depends on the learning strategies applied during teaching on the subject. When selecting a learning strategy, lecturers must consider curriculum objectives and the learning context, including phases of education, environment, and materials. Clearly defining learning outcomes and aligning them with activities ensures IPECP adds value beyond uni-professional learning, while also being less resource-intensive to implement.

## Limitations

The IPEC Core Competencies are complex and often overlap, making it difficult to distinguish them; for instance, ethics is included in all four. Representatives aimed to identify the main intent of each Sub-competency to confirm its inclusion in courses, which may have led to some misidentifications. Discussions, led by the first author, sought to reduce these interpretation differences.

While curriculum mapping provides a static overview of a curriculum, it may not accurately reflect students’ experiences. In this study, curriculum map analysis was strengthened by integrating data from various sources, including representatives’ evaluations of Core Competency inclusion and course content details from university policy documents. These insights could guide future curriculum revisions, and incorporating student feedback might further enhance curriculum applicability.

The mapping detailed course content and associated credits. However, quantifying credits for IPEC Core Competency development was challenging since these competencies were only a part of each course.

## Conclusion

The paper examines curriculum mapping and analysis at a specific university, offering a method for identifying the study year linked to IPEC Core Competencies. This method could aid other institutions in planning integrated IPECP programs that complement existing curricula.

The analysis serves as a foundation for incorporating IPEC competencies into LOOOP, allowing different professions to map their curricula. A key takeaway for faculty is the ability to pinpoint where students in each program can demonstrate IPEC Sub-competencies and how their achievements in IPECP are assessed. This establishes a useful baseline for curriculum planning.

In resource-limited institutions, curriculum mapping can streamline the development of an IPECP program. It helps determine when students are ready to function as part of an interprofessional team in IPECP activities.

The ultimate aim of IPECP is to create a collaborative-ready health workforce that delivers quality healthcare. While creating a new curriculum may be daunting, this study offers a practical approach to integrating IPEC Core Competencies into existing courses, optimizing educational time, and enhancing teamwork skills.

## Data Availability

The data sets are available from the corresponding author on reasonable request.

## Appendix

**Table 3:** Interprofessional Education Collaborative Core Competencies and Sub-competencies (2016) [8]

Code	IPECP Core Competency
Values and Ethics: Work with individuals of other professions to maintain a climate of mutual respect and shared values	
VE1	Place interests of patients and populations at center of IP health care delivery and population health programs and policies, with the goal of promoting health and health equity across the life span
VE2	Respect the dignity and privacy of patients while maintaining confidentiality in the delivery of team based care
VE3	Embrace cultural diversity and individual differences that characterize patients, populations and the health team
VE4	Respect unique cultures, values, roles/responsibilities and expertise of other health professions and the impact of these factors can have on health outcomes
VE5	Work in cooperation with those who receive care, provide care and others who contribute to or support the delivery of prevention and health services and programmes
VE6	Develop a trusting relationship with patients, families and other team members
VE7	Demonstrate high standards of ethical conduct and quality of care in contributions to team-base care
VE8	Manage ethical dilemmas specific to interprofessional patient/population centered care situations
VE9	Act with honesty and integrity in relationships with patients, families, communities and other team members
VE10	Maintain competence in one’s own profession appropriate to scope of practice
Roles and Responsibilities: Use the knowledge of one’s own role and those of other professions to appropriately assess and address the health care needs of patients and to promote and advance the health of populations	
RR1	Communicate one’s roles and responsibilities clearly to patients, families, community members and other professionals
RR2	Recognize one’s limitations in skills, knowledge and abilities
RR3	Engage diverse professionals who complement one’s own professional expertise, as well as associated resources, to develop strategies to meet specific health and healthcare needs of patients and populations
RR4	Explain the roles and responsibilities of other providers and how the team works together to provide care, promote health and prevent disease
RR5	Use the full scope of knowledge, skills and abilities of professionals from health and other fields to provide care that is safe, timely, efficient, effective and equitable
RR6	Communicate with team members to clarify each member’s responsibility in executing components of a treatment plan or public health intervention
RR7	Forge independent relationships with other professions within and outside of the health system to improve care and advance learning
RR8	Engage in continuous professional and interprofessional development to enhance team performance and collaboration
RR9	Use unique and complimentary abilities of all members of the team to optimize health and patient care
RR10	Describe how professionals in health and other fields can collaborate and integrate clinical care and public health interventions to optimize population health
Interprofessional Communication: Communicate with patients, families, communities, and professionals in health and other fields in a responsive and responsible manner that supports a team approach to the promotion and maintenance of health and the prevention and treatment of disease	
CC1	Choose effective communication tools and techniques, including information systems and communication technologies, to facilitate discussions and interactions that enhance team function
CC2	Communicate information with patients, families, community members and health team members in a form that is understandable, avoiding discipline-specific terminology when possible
CC3	Express one’s own knowledge and opinion to team members involved in patient care and population health improvements with confidence, clarity and respect, working to ensure common understanding of information, treatment, care decisions and population health programs and policies
CC4	Listen actively and encourage ideas and opinions of other team members
CC5	Give timely, sensitive, instructive feedback to others about their performance on the team, responding respectfully as a team member to feedback of others
CC6	Use respectful language appropriate for a given difficult situation, crucial conversation or conflict
CC7	Recognize how one’s uniqueness contributes to effective communication, conflict resolution, and positive interprofessional working relationships
CC8	Communicate the importance of teamwork in patient-centered care and population health programmes and policies
Team and teamwork: Apply relationship-building values and the principles of team dynamics to perform effectively in different team roles to plan, deliver, and evaluate patient/population centered care and population health programs and policies that are safe, timely, efficient, effective, and equitable	
TT1	Describe the process of team development and the roles and practices of effective teams
TT2	Develop consensus on the ethical principles to guide all aspects of team work
TT3	Engage health and other professionals in shared patient-centered and population-focused problem solving
TT4	Integrate the knowledge and experience of health and other professions to inform health and care decisions, while respecting patient and community values and priorities /preference for care
TT5	Apply leadership practices that support collaborative practice and team effectiveness
TT6	Engage self and others to constructively manage disagreements about values, roles, goals and actions that arise among health and other professionals and with patients, families and community members
TT7	Share accountability with other professions, patients and communities for outcomes relevant to prevention and health care
TT8	Reflect on individual and team performance for individual as well as team performance improvement
TT9	Use process improvement to increase effectiveness of interprofessional teamwork and team-based services, programs and policies
TT10	Use available evidence to inform effective teamwork and team-based practices
TT11	Perform effectively on teams and in different team roles in a variety of settings
